# Arousal State-Dependent Alterations in Neural Activity in the Zebra Finch VTA/SNc

**DOI:** 10.3389/fnins.2020.00897

**Published:** 2020-08-21

**Authors:** Shin Yanagihara, Maki Ikebuchi, Chihiro Mori, Ryosuke O. Tachibana, Kazuo Okanoya

**Affiliations:** ^1^Graduate School of Arts and Sciences, The University of Tokyo, Tokyo, Japan; ^2^RIKEN Center for Brain Science, Wako, Japan

**Keywords:** sleep, wakefulness, VTA, SNc, dopamine, songbird

## Abstract

Sleep-wake behaviors are important for survival and highly conserved among animal species. A growing body of evidence indicates that the midbrain dopaminergic system is associated with sleep-wake regulation in mammals. Songbirds exhibit mammalian-like sleep structures, and neurons in the midbrain ventral tegmental area (VTA) and substantia nigra pars compacta (SNc) possess physiological properties similar to those in mammals. However, it remains uncertain whether the neurons in the songbird VTA/SNc are associated with sleep-wake regulation. Here, we show that VTA/SNc neurons in zebra finches exhibit arousal state-dependent alterations in spontaneous neural activity. By recording extracellular single-unit activity from anesthetized or freely behaving zebra finches, we found that VTA/SNc neurons exhibited increased firing rates during wakefulness, and the same population of neurons displayed reduced firing rates during anesthesia and slow-wave sleep. These results suggest that the songbird VTA/SNc is associated with the regulation of sleep and wakefulness along with other arousal regulatory systems. These findings raise the possibility that fundamental neural mechanisms of sleep-wake behaviors are evolutionarily conserved between birds and mammals.

## Introduction

Midbrain dopaminergic neurons in the ventral tegmental area (VTA) and substantia nigra pars compacta (SNc) play an important role in a wide variety of behaviors such as learning, motivation, and movement (Berke, 2018). However, the relationship between VTA/SNc and sleep-wakefulness has been relatively overlooked. Early *in vivo* electrophysiological studies in rats (Miller et al., 1983) and cats (Trulson and Preussler, 1984) showed that dopaminergic neurons in the VTA/SNc have similar firing rates during sleep and wakefulness, while a later study revealed enhanced burst firing during REM sleep (Dahan et al., 2007). In contrast, non-dopaminergic neurons in the VTA/SNc showed increased firing rates during wakefulness and REM sleep (Miller et al., 1983; Lee et al., 2001). More recently, a growing body of evidence supports the notion that VTA dopaminergic and non-dopaminergic neurons regulate sleep-wake behaviors in rodents. VTA dopaminergic neurons exhibit higher calcium activity during wakefulness and REM sleep than during non-REM sleep (Eban-Rothschild et al., 2016), and activation of dopaminergic neurons by optogenetics or chemogenetics induces wakefulness (Eban-Rothschild et al., 2016; Taylor et al., 2016; Oishi et al., 2017). Besides, VTA GABAergic neurons show high calcium activity during wakefulness and REM sleep (Yu et al., 2019; Eban-Rothschild et al., 2020), and chemogenetic activation of VTA GABA and glutamate neurons induce changes in arousal states (Yu et al., 2019). Although sleep-wake behaviors are evolutionarily conserved among animal species, much of what we know has been learned from studies in mammals. Thus, the relationship between VTA/SNc neurons and sleep-wake regulation in non-mammalian vertebrates such as birds remains unknown.

Songbirds provide a unique opportunity to study the relationship between dopaminergic system and sleep-wake regulation. Birds exhibit sleep structures similar to mammals (Blumberg and Rattenborg, 2017) and mammalian-like sleep features (such as slow-wave sleep, intermediate sleep, and REM sleep) have been demonstrated in zebra finches (Low et al., 2008). In addition, the midbrain dopaminergic system has been relatively well-characterized in zebra finches. The VTA/SNc contains dopaminergic and non-dopaminergic neurons, which share many physiological properties and anatomical connections with those in mammals (Lewis et al., 1981; Gale and Perkel, 2006). Recent electrophysiological studies show that basal ganglia-projecting dopaminergic neurons in the VTA encode performance errors (Gadagkar et al., 2016), and manipulation of dopaminergic activity affects song learning (Hisey et al., 2018; Xiao et al., 2018). These studies highlight the significance of the VTA dopaminergic system in sensorimotor learning in songbirds. Nevertheless, it remains uncertain whether VTA/SNc neurons in songbirds are associated with sleep-wake regulation. Given the importance of sleep-wake regulation and anatomical homologies between mammalian and avian brains, comparing the neural activity in the VTA/SNc between birds and mammals is critical to uncover the common neural mechanisms underlying the regulation of sleep and wakefulness. We thus examined whether VTA/SNc neurons in zebra finches exhibit arousal state-dependent alterations in neural activity by recording extracellular single-unit activity from anesthetized and freely behaving conditions.

## Materials and Methods

### Animals

Male zebra finches were obtained from our breeding colony (*n* = 6) or purchased from a local supplier (*n* = 2). Birds were kept on a 14L:10D photoperiod. Food and water were available *ad libitum*. Data were acquired between 9:30 a.m. and 8:30 p.m. (light-phase: 7:00 a.m. to 9:00 p.m.). On each bird, 2–5 recording sessions were made (3 ± 1.3 sessions, mean ± SD, *n* = 8 birds, 2 anesthetized and 6 free behaving conditions) over 1–5 recording days (2.6 ± 1.6 days, mean ± SD). For electrophysiological recordings in anesthetized conditions, two adult birds from a local supplier (>120 days post-hatch, exact age unknown) were used. For electrophysiological recordings in free behaving conditions, six birds from our breeding colony (recording onset: 56–86 days post-hatch, 64 ± 11.3 days, mean ± SD) were used. Single-unit recordings in free behaving conditions were performed from both juvenile (<90 days, 56–87 days post-hatch, 33 single-units from 6 birds) and adult period (>90 days, 91–101 days post-hatch, 3 single-units from 1 bird). Since there were no differences between the juvenile and adult period data obtained from free behaving birds, both data sets were pooled for the subsequent analysis. All experiments were approved by the animal experimentation committee at the University of Tokyo and performed in accordance with the established guidelines.

### Surgery and Electrophysiological Recordings

General surgical procedures and electrophysiological recordings were described in a previous report (Yanagihara and Hessler, 2012). Single-unit activity from VTA/SNc under isoflurane anesthesia was recorded extracellularly in head-restrained adult birds (*n* = 2). In brief, birds were anesthetized with 1.5% isoflurane and placed in a stereotaxic apparatus (Narishige) on top of a heating pad. Lidocaine (2%, Maruishi Pharmaceutical) was applied to the scalp before the incision was made. Small craniotomies were made, and a tungsten electrode (3 MΩ, Microprobe) or glass capillary filled with Fluoro-Ruby (2.5%, AG335 Merck Millipore) was lowered into the brain using motorized manipulator (MC-5B, National Aperture Inc.). Extracellular neuronal signals were amplified (10,000-fold), band-pass filtered (0.5–9 kHz), digitized (40 kHz) with a Plexon recorder, and stored on a PC as data. Extracellular single-unit and local field potential (LFP) activity was recorded from VTA/SNc in freely behaving birds (*n* = 6). A manually movable microdrive attached to four bundles of tetrode wire and reference wire (12.5 μm in diameter, RO800, Sandvic) was chronically implanted while juvenile zebra finches were anesthetized with 1–1.3% isoflurane in a stereotaxic apparatus. Stereotaxic coordinates used for VTA/SNc were as follows; anterior: 0.8–1.0 mm, lateral: 0.5–0.8 mm, depth: 5.7–6.7 mm from the bifurcation of the sagittal sinus, head angle: 28 degrees. After recovery from the surgery, birds were kept in a recording chamber (30 × 20 × 25 cm), which was placed in a sound attenuation box (50 × 40 × 40 cm), and single-unit and LFP activity were recorded while they were awake and freely behaving. Extracellular neuronal signals were amplified (10,000-fold), band-pass filtered (0.5–9 kHz), and digitized (40 kHz), and LFP signals were amplified (1,000-fold), band-pass filtered (0.7–170 Hz), and digitized (20 kHz) with a Plexon MAP system. To monitor behavioral states, a digital video camera was placed in the sound attenuation box and video signals were recorded synchronously with neural data acquisition (CinePlex Behavioral Research System, Plexon). Both neural and video data were stored on a PC.

### Analysis of Electrophysiological Data

Spike sorting was performed using an off-line sorter (Plexon), and well-isolated single-units were analyzed with MATLAB (MathWorks). For each single-unit, spike width was calculated as half-width of first negative deflections of mean of 20 spike waveforms. To compare the difference in firing rates between isoflurane-induced anesthetized and awake states, mean firing rates were calculated for each neuron using 30 s during anesthetized or awake periods. A bird was considered awake when the bird’s eyes were kept open for at least 30 s after the isoflurane was turned off. To quantify burst firing, spikes were determined as a burst when the inter-spike interval (ISI) was lower than a threshold (<6 ms) and ISI-based bursting index, the proportion of the burst, was calculated (Simonnet and Brecht, 2019). Variability of firing was quantified using the coefficient of variation (CV) of ISI (CV = SD/mean).

For data obtained from the freely behaving condition, inspection of video and LFP signals was used to assess the birds’ arousal states. Periods of sleep were determined by immobility, eye-closing, and slow-wave activity in LFP signals (Figures 3B,D). In all other periods, birds were determined to be awake. Spectral power of LFP for each bird was calculated in mV^2^/Hz for 3-s windows using MATLAB toolbox EEGLAB (Brunner et al., 2013). Mean power spectral density (PSD) of 10 epochs of awake or sleep period was separately calculated for each bird, and averaged over all birds (*n* = 6 free behaving birds, Figure 3G). Mean awake firing rates, ISI-based bursting index, and CV of ISI were calculated from a 30-s data segment during an awake period where birds were neither vocalizing nor eating/drinking. Multiple units were recorded per bird. Since the activity of these units are unlikely to be independent from each other, the values for individual birds were averaged together for the purpose of statistical analysis (Figure 4C). To compare the difference in firing rates between sleep and awake states, nonparametric Wilcoxon signed-rank test for paired samples was used for statistical testing. The significance level was set at α = 0.05. In addition to the above analysis, narrow-spike units and wide-spike units data were separately analyzed (Figure 4D). In the case of narrow-spike data, units with a spike half-width less than 0.12 ms were included. In the case of broad-spike data, units with a spike half-width more than 0.12 ms and firing rate during sleep less than 15 Hz were included. Since there were no differences between the neural data acquired from VTA and SNc, both data sets were pooled for the statistical analysis.

### Anatomical Verification of Recording Sites

**FIGURE 1:**
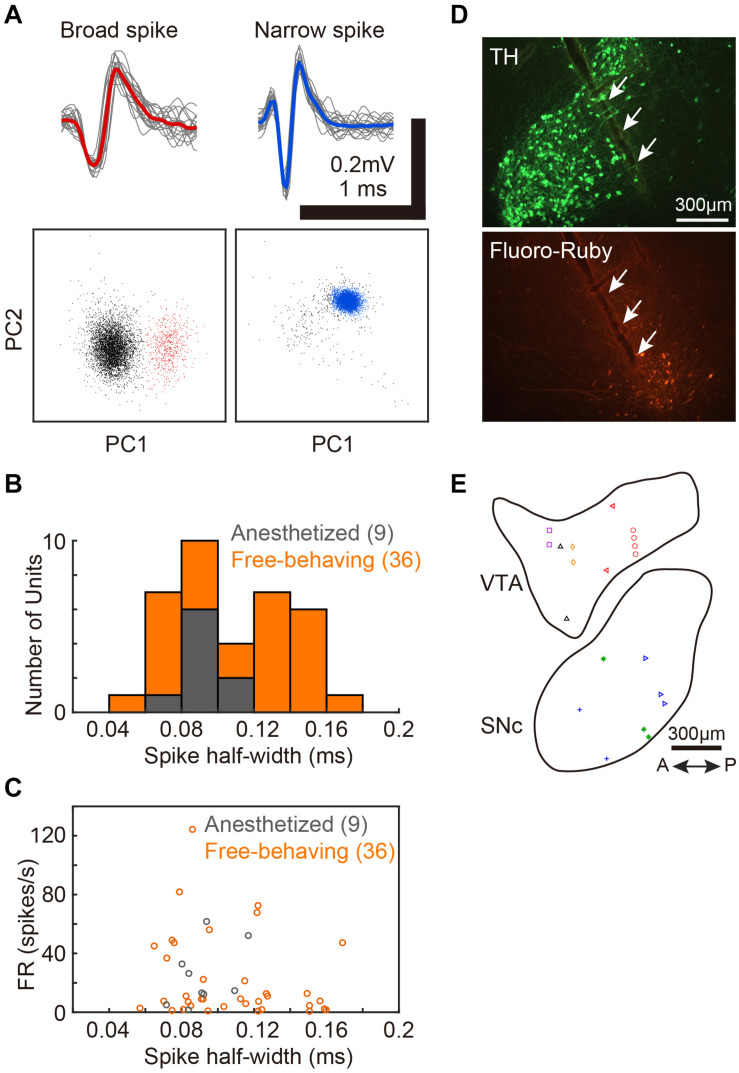
Single-unit recordings from VTA/SNc and the positions of recording sites. **(A)** Two distinct spike waveforms (top) and the spike clusters (bottom) from 2 single-units in VTA. Broad spike (red, left) and narrow spike (blue, right) units. Red and blue lines represent mean spike waveform of each single-unit. The gray lines represent the single spikes (20 spike waveforms). In the bottom panels, colored dots (red and blue) indicate extracted spikes as a single-unit. Gray dots indicate noise cluster. **(B)** Distributions of spike half-width of all single-units recoded from anesthetized (gray histogram, 9 units, 2 birds) and free-behaving (orange histogram, 36 units, 6 birds) conditions. **(C)** Spontaneous firing rates of each VTA/SNc unit during awake state is plotted against the spike half-width. Gray and orange circles denote single-unit data from anesthetized and free-behaving conditions, respectively. **(D)** Histological verification of electrophysiological recording sites. Tyrosine hydroxylase (TH) immunostaining (top) and injection sites of Fluoro-Ruby (bottom). White arrows indicate the track of the recording electrode in SNc. **(E)** Recording sites from all birds (2 anesthetized and 6 free-behaving conditions) are displayed in the example sagittal sections. Same symbols (color/shape) denote recordings from the same bird. Multiple single-units (2–6 units) were simultaneously recorded at 10 out of the 20 recording sites.

After electrophysiological recordings, electrical lesions were made (20 μA, 20 s, Stimulus Isolator A365, WPI) or Fluoro-Ruby was deposited using an iontophoresis pump (BAB-501, Kation Scientific). The birds were deeply anesthetized with an overdose of pentobarbital sodium (Somnopentyl, Kyoritsu Seiyaku) and perfused with 4% paraformaldehyde (PFA), and brains were dissected out. The brains were post-fixed overnight in 4% PFA followed by 30% sucrose in phosphate-buffered saline. Sagittal brains sections (40 μm in thickness) were made with a freezing microtome (ROM-380, Yamato Kohki Industrial). Midbrain dopaminergic neurons were stained with an antibody against tyrosine hydroxylase (TH, MAB318, Merck Millipore), and the recording electrode track or injection site of Fluoro-Ruby was verified (Figure 1D).

## Results

**FIGURE 2:**
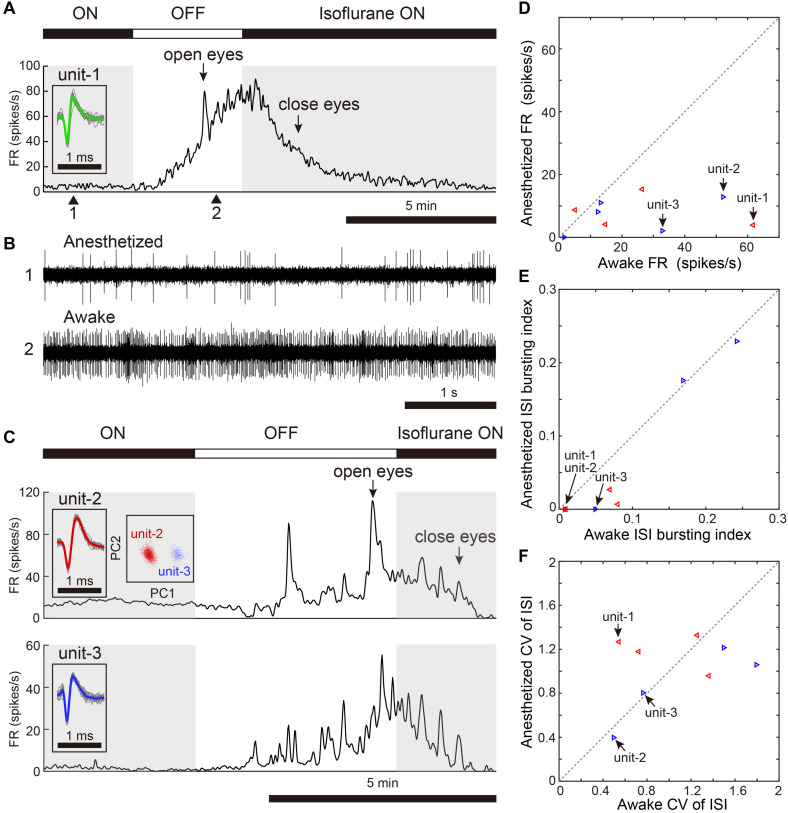
Changes in spontaneous neural activity across isoflurane-anesthetized and wake states. **(A)** Representative spike rate histogram of a single VTA neuron (unit-1) under isoflurane anesthesia. The top bar displays periods when isoflurane anesthesia is turned on (black) and off (white). Gray shadings denote the anesthetized period. Arrows indicate when birds opened and closed their eyes. Inset shows spike waveforms. **(B)** Example of raw spike traces under anesthetized (1, top) and awake (2, bottom) states. **(C)** Representative spike rate histogram of simultaneously recorded 2 single SNc neurons (unit-2, -3) under isoflurane anesthesia. Insets show spike waveforms and spike clusters. **(D)** Comparison of mean spontaneous firing rates between anesthetized and awake (unanesthetized) states, *n* = 9 neurons, 2 birds. **(E)** Comparison of ISI-based bursting index, proportion of ISIs <6 ms, between anesthetized and awake states. **(F)** Comparison of CV (coefficient of variation) of ISI between anesthetized and awake states. The data shown in **(A,C)** (unit-1-3) are indicated by arrows in the scatter diagram **(D–F)**. Same symbols (color/shape) in **(D–F)** are data from the same bird and correspond to the histological data in Figure 1E.

We assessed activity in VTA/SNc neurons across sleep-wake states by electrophysiological recordings under isoflurane anesthesia or in freely behaving conditions. Consistent with the previous studies (Gale and Perkel, 2006; Kearney et al., 2019), we observed broad spike and narrow spike neurons in the zebra finch VTA/SNc (Figure 1). Broad spike neurons tended to show low spontaneous firing rates, while narrow spike neurons showed variable firing rates including both low and high spontaneous firing rates (Figure 1C). During single-unit recordings from head-restrained zebra finches under anesthesia, isoflurane was briefly turned off to examine whether the neural activity was altered (Figure 2). When isoflurane was turned off for a few minutes, the firing rate of the neurons gradually increased prior to eye opening (Figures 2A,C). After turning on isoflurane, on the other hand, the firing rate gradually decreased prior to eye closing. When comparing neural activity across anesthetized and awake states, most neurons showed higher firing rates during awake state (Figure 2D). To quantify burst firing activity, we defined ISI-based bursting index calculated as proportion of short ISIs (<6 ms). When comparing the bursting index across anesthetized and awake states, some neurons showed higher bursting index during awake state (Figure 2E). To compare the firing variability between anesthetized and awake states, we calculated the coefficient of variation (CV) of ISI and there was no difference in the firing variability (Figure 2F).

**FIGURE 3:**
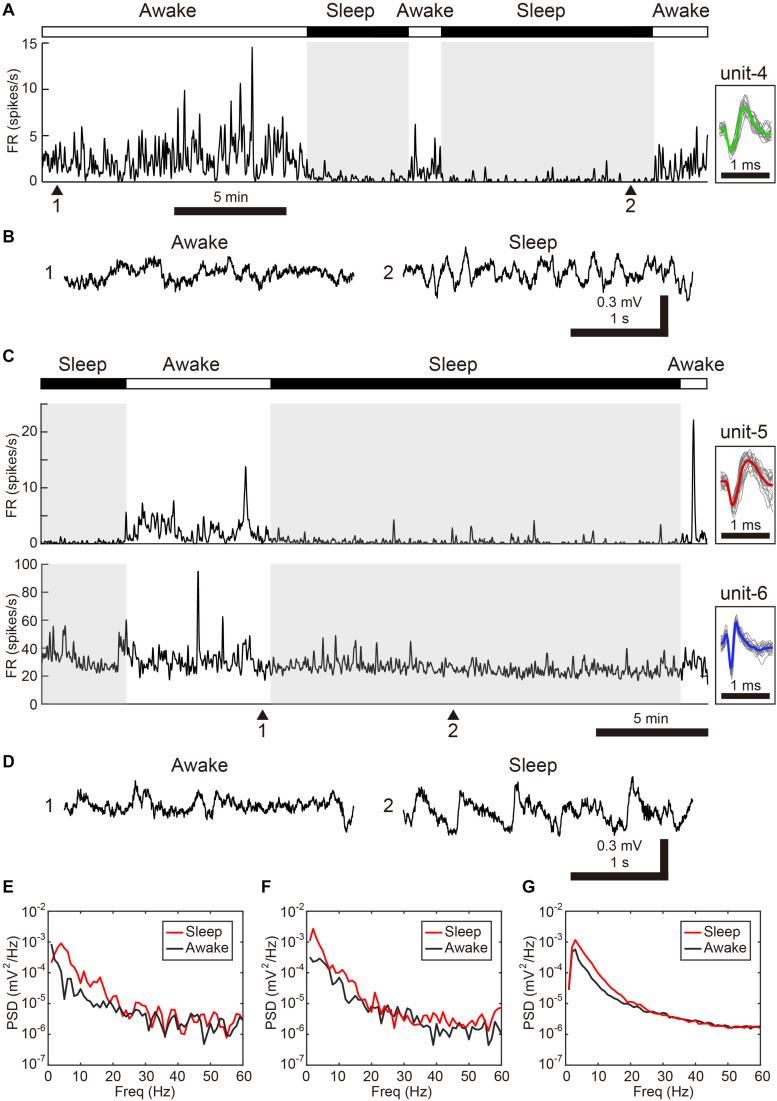
Changes in spontaneous neural activity across sleep-wake states in the freely behaving condition. **(A)** Representative spike rate histogram of a single SNc neuron (unit-4) during natural sleep-wake transitions. The top bar displays awake (white) and sleep (black) periods. Gray shadings denote sleep periods. Inset shows spike waveforms. **(B)** Example of LFP traces under awake (1) and sleep (2) periods. Note slow-wave activity during sleep. **(C)** Representative spike rate histogram of simultaneously recorded 2 single VTA neurons (unit-5, -6) during natural sleep-wake transitions. **(D)** Example of LFP traces under awake (1) and sleep (2) periods. **(E)** Power spectral density (PSD) for samples in 3-s windows (awake: 1, sleep:2) shown in **(B)**. **(F)** PSD for samples (awake: 1, sleep:2) shown in **(D)**. **(G)** Mean PSD across all 6 free behaving birds for awake and sleep periods.

**FIGURE 4:**
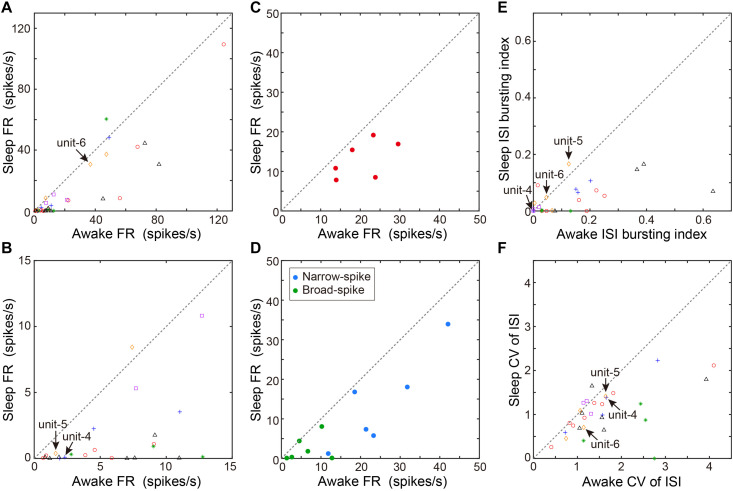
Comparison of spontaneous neural activity between sleep and awake states. **(A)** Comparison of firing rates between awake and sleep states (*n* = 36 neurons, 6 birds). Each symbol indicates data from a single unit. Same symbols (color/shape) are from the same bird and correspond to the histological data in Figure 1E. **(B)** The low firing rate portion (0–15 Hz) of the same data as in **(A)** are displayed for clarity. **(C)** Comparison of mean firing rate of all units recorded from individual birds (*n* = 6 birds) between sleep and awake states. Each symbol indicates data from a single bird. **(D)** Comparison of mean firing rate of narrow-spike units (blue circles) or broad-spike units (green circles) recorded from individual birds (*n* = 6 birds) between sleep and awake states. Each symbol indicates data from a single bird. **(E)** ISI-based bursting index (proportion of ISIs < 6 ms) during awake state and sleep state. Each symbol indicates data from a single unit. **(F)** CV of ISI during awake and sleep state. Each symbol indicates data from a single unit. The data shown in Figures 3A,C (unit-4-6) are indicated by arrows in each scatter diagram.

We next examined whether VTA/SNc neurons also exhibit arousal state-dependent alterations in neural activity during natural sleep-wake transitions. Since zebra finches, especially juvenile birds, often engage in daytime naps (Margoliash and Schmidt, 2010), we took advantage of this natural sleep-wake transition. We recorded multiple single-units and local field potentials (LFPs) from VTA/SNc in freely behaving juveniles during sleep-awake transitions (Figure 3). In the daytime, juvenile zebra finches showed brief episodes where they transitioned from awake to asleep, and typically each sleep episode lasted for several minutes (Figures 3A,C). States of arousal were determined by both LFP activity and video signals. Consistent with previous studies in birds (Hahnloser et al., 2006; Low et al., 2008; Yanagihara and Hessler, 2012; van der Meij et al., 2019), LFPs exhibited slow-wave activity during sleep (Figures 3B,D). The LFP signals showed large low-frequency power (<20 Hz) during sleep, while high-frequency power (>20 Hz) during sleep and awake states were indistinguishable (Figures 3E–G). During natural sleep-wake transitions, we observed that VTA/SNc neurons exhibited alterations in their firing rate depending on the arousal states (Figures 3A,C). Overall, most of the VTA/SNc neurons exhibited higher firing rates in awake state than in slow-wave sleep (Figures 4A,B). We averaged together the firing rate of all units recorded from individual birds, and compared between sleep and awake state. We found that VTA/SNc neurons showed higher spontaneous firing rates in the awake than sleep state (Figure 4C, sleep firing rate = 13.1 ± 1.9, awake firing rate = 20.4 ± 2.6, mean ± s.e.m., Wilcoxon signed-rank test, *p* = 0.0313, *n* = 6 birds). We further analyzed broad-spike and narrow-spike units separately, and compared between sleep and awake states (Figure 4D). In narrow-spike unit case, we found significant difference between sleep and wake states (Figure 4D, blue circles, sleep firing rate = 13.9 ± 4.8, awake firing rate = 24.8 ± 4.4, mean ± s.e.m., *p* = 0.0313, *n* = 6 birds). In broad-spike unit case, we also found significant difference between sleep and awake states (Figure 4D, green circles, sleep firing rate = 2.5 ± 1.3, awake firing rate = 6.4 ± 1.8, mean ± s.e.m., *p* = 0.0313, *n* = 6 birds). When comparing bursting activity and firing variability across sleep and awake states, most of the VTA/SNc neurons exhibited higher bursting activity and firing variability in awake state (Figures 4E,F), though the bursting index did not reach statistical significance (sleep bursting index = 0.04 ± 0.01, awake bursting index = 0.13 ± 0.05, mean ± s.e.m., *p* = 0.1563, *n* = 6 birds, sleep CV of ISI = 1.04 ± 0.09, awake CV of ISI = 1.59 ± 0.16, mean ± s.e.m., *p* = 0.0313, *n* = 6 birds). These findings demonstrate that spontaneous activity in VTA/SNc neurons change across sleep-wake transitions with increased firing rates during wakefulness.

## Discussion

In this study, we found that VTA/SNc neurons in zebra finches exhibit arousal state-dependent alterations in neural activity. In both isoflurane-induced anesthesia and natural sleep-awake transitions, most VTA/SNc neurons showed increased spontaneous firing rates during wakefulness and a reduction in firing rates during slow-wave sleep. These results suggest that VTA/SNc neurons may play a role in regulating sleep-wake transitions. To the best of our knowledge, this is the first electrophysiological evidence of arousal state-dependent alterations in neural activity in the songbird VTA/SNc.

Zebra finch VTA/SNc contains dopaminergic and non-dopaminergic neurons, and shares physiological properties similar to those in mammals (Gale and Perkel, 2006). Dopaminergic neurons in both zebra finches and rodents have relatively broad (long duration) action potential waveforms and show slow spontaneous firing rates (<10 Hz), while non-dopaminergic neurons have relatively narrow (short duration) action potential waveforms and show variable or faster spontaneous firing rates (Miller et al., 1983; Gale and Perkel, 2006; Kearney et al., 2019). Consistent with these previous studies, we also observed both broad and narrow spike neurons (Figure 1). Since our *in vivo* electrophysiological recordings were made extracellularly, we cannot definitively identify the cell types that were recorded. Despite this limitation, we observed that some neurons displayed dopaminergic characteristics (slow spontaneous firing rates and broad spike waveforms, Figure 3, unit-4 and unit-5), and others displayed non-dopaminergic characteristics (fast spontaneous firing rates and narrow spike waveforms, Figure 2, unit-1-3, Figure 3, unit-6). Accordingly, the data we obtained from zebra finch VTA/SNc most likely includes both dopaminergic and non-dopaminergic neurons. Importantly, most of the neurons we examined in this study consistently showed increased firing rates, bursting activity, and firing variability during wakefulness compared to slow-wave sleep, thus suggesting that both dopaminergic and non-dopaminergic neurons are involved in sleep-wake regulation. In this study, however, we did not examine the relationship between VTA/SNc activity and REM sleep, because our neural recordings under freely behaving conditions were made during daytime naps and we did not observe REM sleep during such short sleep periods. Since VTA dopaminergic and non-dopaminergic neurons in rodents exhibit enhanced activity during REM sleep as well as wakefulness (Lee et al., 2001; Dahan et al., 2007; Eban-Rothschild et al., 2016, 2020; Yu et al., 2019), it will be important for future studies to examine whether VTA/SNc neurons in zebra finches show similar enhanced firing activity during REM sleep. Recent studies in mammals (Miller et al., 1983; Lee et al., 2001; Eban-Rothschild et al., 2016, 2020; Taylor et al., 2016; Oishi et al., 2017; Yu et al., 2019) and insects (Kume et al., 2005) demonstrate that the midbrain dopaminergic system is involved in the regulation of sleep-wake transitions. Consistent with this, our results show alterations in neuronal activity across sleep and wakefulness in the songbird VTA/SNc, thus suggesting that the role of the dopaminergic system in sleep-wake regulation is evolutionarily conserved.

Sleep is highly conserved among most animals, and a growing body of evidence demonstrates the importance of sleep in memory consolidation and learning in many animal species (Deregnaucourt et al., 2005; Klinzing et al., 2019; van der Meij et al., 2020). In juvenile zebra finches, frequent transitions between sleep and wakefulness during the daytime is assumed to play a role in vocal learning (Margoliash and Schmidt, 2010). In this study, we observed such daytime sleep-wake transitions in juveniles and spontaneous activity in VTA/SNc neurons was tightly linked to arousal state at the single neuron level. One possible function of VTA/SNc neurons in juveniles may be an arousal switch from wakefulness to an off-line sleep mode in the daytime, leading to memory consolidation after hearing song from a tutor bird or after vocal practice. In support of this idea, neural replay or enhanced spontaneous burst activity has been demonstrated in the song nuclei during sleep (Dave and Margoliash, 2000; Hahnloser et al., 2006; Shank and Margoliash, 2009; Yanagihara and Hessler, 2012), and those brain areas receive dopaminergic and non-dopaminergic inputs from the VTA/SNc (Lewis et al., 1981; Appeltants et al., 2000, 2002). In concert with other neuromodulatory systems such as noradrenaline (Castelino and Schmidt, 2010), the VTA/SNc may change brain states to facilitate leaning and sensory processing. Further studies to manipulate VTA/SNc neuronal activity (Xiao et al., 2018) during sleep will be necessary to understand the functional relationships among the dopaminergic system, arousal regulation, and learning. In conclusion, our study shows that songbird VTA/SNc neurons display arousal-dependent changes in spontaneous activity, suggesting that the midbrain dopaminergic system plays an evolutionarily conserved role in sleep-wake regulation.

## Data Availability Statement

Datasets generated for this study are available from the corresponding author on reasonable request.

## Ethics Statement

The animal study was reviewed and approved by the animal experimentation committee at the University of Tokyo.

## Author Contributions

SY conducted the experiments, analyzed the data, and wrote the original draft. MI, CM, RT, and KO reviewed the manuscript and provided critical input. KO supervised the research. All authors contributed to the article and approved the submitted version.

## Conflict of Interest

The authors declare that the research was conducted in the absence of any commercial or financial relationships that could be construed as a potential conflict of interest.
